# A Hiplot‐based web service for cold atmospheric plasma high‐throughput data integration and analysis on breast cancer

**DOI:** 10.1002/imt2.70045

**Published:** 2025-05-25

**Authors:** Xiaofeng Dai, Mingjie Wang, Yang Liu

**Affiliations:** ^1^ National Local Joint Engineering Research Center for Precision Surgery & Regenerative Medicine, Shaanxi Provincial Center for Regenerative Medicine and Surgical Engineering The First Affiliated Hospital of Xi'an Jiaotong University, Xi'an Jiaotong University Xi'an China; ^2^ Department of Gastroenterology, Ruijin Hospital Shanghai Jiaotong University, School of Medicine Shanghai China

**Keywords:** acetylome, breast cancer, cold atmospheric plasma, Hiplot, phosphorylome, proteome, whole transcriptome

## Abstract

Incremental evidence on the effect of cold atmospheric plasma (CAP) in specifically killing transformed cells and advances in sequencing technologies at multiple omics have led to the demand of in‐depth exploration on the mechanisms of action driving the potency of CAP against cancer cells at the molecular level. However, high‐throughput data detailing the effect of CAP on cancer cells is lacking, let alone the corresponding database and analytical tool. Here, we sequenced the whole transcriptome, proteome, phosphorylome, acetylome, and lactylome of transformed cells in response to CAP using breast cancer cells as the disease model; and advanced our previously developed Hiplot platform by establishing a focus‐driven tumor‐specific module, namely CAP medicine in breast cancer (CAPmed‐BC) (https://capbc.hiplot.com.cn). CAPmed‐BC is the first multi‐omics data resource in plasma medicine for analyzing the treatment response of breast cancer cells to CAP. It can analyze each type of omics data regarding differentially expressed biomarkers, expression landscape, gene ontology analysis, pathway interpretation, gene set enrichment analysis, and protein‐protein interaction network. It can also interrogate the dynamic fluctuation, functional activity, and metabolic vulnerability of cancer cells in response to CAP by combinatorially analyzing omics at multiple carefully defined dimensions. We also built in a visualization module to support users for producing personalized graphs via adjusting parameters. We believe that CAPmed‐BC will become a valuable resource for characterizing the outcome of CAP on breast cancers at the omics and molecular levels, and make considerable contributions to both plasma medicine and oncology.

## Highlights

- Cold atmospheric plasma (CAP) represents an emerging onco‐therapeutics that can specifically ablate cancer cells without harming their healthy peers via redox perturbation.
- CAP medicine in breast cancer (CAPmed‐BC) is the first reservoir of multi‐level omics data including whole transcriptome, acetylome, lactylome, proteome, phosphorylome that records the responses of different types of breast cancer cells to CAP treatment.
- CAPmed‐BC can be used to capture the dynamics, functionality and metabolism of triple negative breast cancer cells in response to CAP treatment.
- CAPmed‐BC is unique in interrogating cell metabolic alterations through coupled analysis of acetylome and lactylome.

## Graphical abstract

Cold atmospheric plasma (CAP) represents an emerging onco‐therapeutics that can specifically ablate cancer cells without harming their healthy peers via redox perturbation. CAP medicine in breast cancer (CAPmed‐BC) is the first reservoir of multi‐level omics data including whole transcriptome, acetylome, lactylome, proteome, phosphorylome that records the responses of different types of breast cancer cells to CAP treatment. Being the first functional module of Hiplo, CAPmed‐BC can be used to capture the dynamics, functionality, and metabolism of triple negative breast cancer cells in response to CAP treatment, and is unique in interrogating cell metabolic alterations through coupled analysis of acetylome and lactylome.

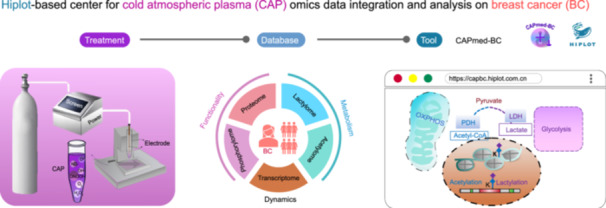

## INTRODUCTION

Breast cancer (BC) is the most prevalent malignancy among women that is featured with high heterogeneity. According to the surface expression of estrogen receptor, progesterone receptor, and human epithelial receptor 2 (HER2), BC can be grouped into four subgroups, that is, luminal A, luminal B, HER2 positive, and triple negative BC (TNBC). Among these subtypes, the TNBC subtype has a poor clinical outcome and currently relies primarily on chemotherapy that is associated with non‐negligible adverse effects. Thus, there is a clinical need for establishing innovative technologies to effectively resolve TNBC with desirable safety.

Cold atmospheric plasma (CAP) is a type of plasma that belongs to the fourth state of matter besides solid, liquid, and gas. It is primarily comprised of reactive oxygen and nitrogen species (RONS) such as hydroxyl radical (•OH), superoxide (O_2_^−•^), singlet oxygen (_1_O^2^), hydrogen peroxide (H_2_O_2_), ozone (O_3_), nitric oxide (•NO), nitrogen dioxide (•NO_2_), dinitrogen tetroxide (N_2_O_4_), nitrogen trioxide (NO_3_), nitrous oxide (N_2_O), and peroxynitrite (ONOO^−^) [1, 2]. While short‐lived species such as •OH, O_2_^−•^, _1_O^2^ have transient life‐spans, long‐lived species such as H_2_O_2_ and O_3_ have relatively long‐term activities. These species collectively interact with the cell surface to initiate and relay intracellular signaling to achieve specific killing of transformed cells without harming their healthy peers [3, 4]. This unique trait of CAP in ablating cancer cells may be attributable to the differential redox levels and distinct membrane features between cells attracted at the physiological and pathological states. The higher level of RONS produced from the electron transport chain during mitochondria respiration [5] and typically loss‐of‐function of the antioxidant system [6] in transformed cells make these chaotic cells more vulnerable to oxidative stress. In addition, cancer cells possess, in general, abundant aquaporins [7] and lower level of cholesterol [8, 9], improving the sensitivity of transformed cells to CAP than normal cells. Incremental preclinical evidence has suggested the efficacy and safety of CAP in treating various types of cancers [10, 11, 12, 13, 14, 15, 16, 17, 18, 19, 20, 21] including that of breast [22, 23, 24, 25, 26, 27, 28, 29, 30, 31]. Clinically, CAP has been used to secure the life of a 75‐year advanced pancreatic cancer patient in 2016, and the life of a 33‐year relapsed peritoneal sarcoma patient in 2019 [32]. A clinical investigation involving six advanced neck and head cancer patients documented the overall benefits of using CAP for cancer treatment [33], and a 2‐year phase I trial (NCT04267575) reported prolonged survival of 17 out of 20 advanced solid cancer patients recruited [34, 35]. These encouraging preclinical and clinical results have unanimously supported the use of CAP in oncology. Yet, its clinical translation process is substantially hindered due to a lack of complete understanding on the mechanisms‐of‐action driving its dose‐dependent potency [36, 37, 38, 39] in resolving cancers.

**FIGURE 1:**
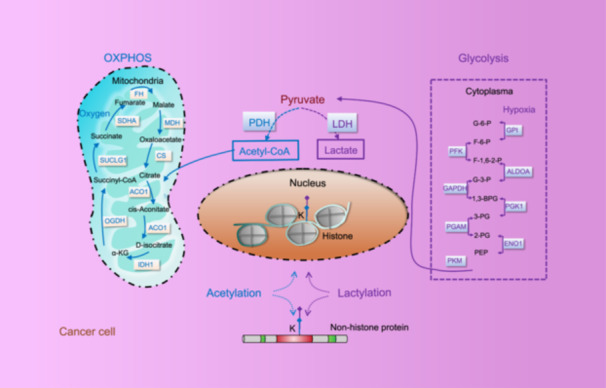
Illustrative diagram showing the rational for gaining metabolic clues from combining the acetylome and lactylome using cold atmospheric plasma medicine in breast cancer (CAPmed‐BC). Differences on the metabolic modes can be reflected from the competitive lysine acetylation and lactylation modification patterns. Given that lysine acetylation and lactylation share the same catalytic enzyme, p300, the amounts of substrates (acetyl‐CoA, lactate) dictate their post‐modification levels. Define the ratio between glycolysis and oxidative phosphorylation as ‘R’, the higher R is the more lactates cells produce and the higher levels of lysine lactylation occur, and vice versa.

With our incremental understandings on the diversity of omics and their functionalities, as well as rapid advances in sequencing technologies, there is an urgent demand on interrogating cellular behaviors in response to CAP towards in‐depth understandings on orchestrated signalings across multiple omics. Here, we sequenced the landscapes of five omics, that is, the whole transcriptome, proteome, phosphorylome, lactylation, and acetylation. The whole transcriptome data including mRNA, miRNA, lncRNA, circRNA are included to capture the dynamic response of TNBC cells to CAP treatment, where profiles at 1 h and 8 h post‐CAP treatment were included to reflect the short‐ and long‐term outcome of transformed cells. Proteome and phosphorylome are sequenced to investigate the functional activity of TNBC cells in response to CAP perturbation. Since lactylation and acetylation compete for epigenetic modification of lysines and mark the levels of lactates and acetyl‐CoA [40], lactylome and acetylome are encompassed to identify vital molecules marking metabolic switch of transformed cells that are vulnerable to CAP treatment and explore clues implicating metabolic rewiring of TNBC cells in response to CAP treatment (Figure 1).

We have previously established the Hiplot platform (https://hiplot.com.cn) to help users quickly analyze publicly available or self‐produced high‐throughput data [41]. Given the incremental demand on tumor omics data enquiry, tools such as BEST (https://rookieutopia.hiplot.com.cn/app_direct/BEST/) [42] have evolved to support tumor‐associated investigation. In response to the increasing significance of translational medicine, we actively advanced Hiplot by integrating multi‐omics data in breast cancer treated with CAP and establishing a tumor‐specific database and analytical module, namely CAPmed‐BC (cold atmospheric plasma medicine in breast cancer) database (https://capbc.hiplot.com.cn), within this robust community ecosystem.

CAPmed‐BC can be used for easy and quick identification of molecules rendering TNBC cells vulnerable to CAP treatment. Specifically, CAPmed‐BC can be used to analyze each type of omics data regarding differential expression, gene ontology (GO) enrichment, Kyoto Encyclopedia of Genes and Genomes (KEGG) pathway analysis, gene set enrichment analysis (GSEA), and protein–protein interaction (PPI) network. It can also be employed to interrogate the dynamic fluctuation, functional activity and metabolic plasticity of TNBC cells in response to CAP through integrative analysis of omics at multiple carefully defined dimensions. Molecules identified may implicate mechanisms‐of‐action potentiating the anticancer efficacy of redox perturbation (i.e., not limited to CAP) in oncology (i.e., not restricted to TNBC), and contribute to the design of innovative regimens for resolving cancers.

## RESULTS AND DISCUSSION

### Data overview

#### Data type

The CAPmed‐BC website contains a comprehensive collection of omics data from breast cancer cells treated with CAP. Omics covered in this database include whole transcriptome, proteome, phosphorylome, lactylome, and acetylome.

**FIGURE 2:**
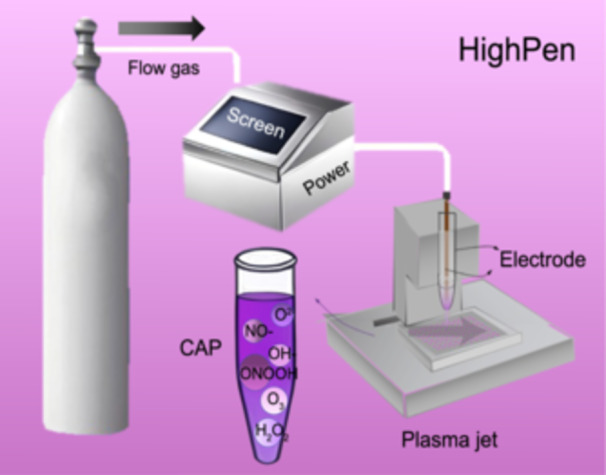
Illustrative diagram showing the infrastructure of HighPen and the procedure of cold atmospheric plasma (CAP) preparation. HighPen is composed of a controlled power supply, gas cylinder, and a plasma jet that can work in a controllable high‐throughput fashion. CAP‐activated medium is used and prepared by treating the desirable medium with HighPen for a specified duration.

The whole transcriptome includes mRNA, lncRNA, and circRNA sequencing data, which provide information on the gene expression profiles of breast cancer cells in response to CAP (Figure 2) at 1 h and 8 h, respectively. The whole transcriptome data (including mRNA, miRNA, lncRNA, and circRNA) is included to capture the dynamic response of breast cancer cells to CAP treatment, where 1 h and 8 h transcriptome profiles were used to represent short‐term and long‐term responses of transformed cells to CAP‐imposed external perturbations.

The proteome and phosphorylome data include protein quantification and modification sites that reveal the amount and activity of proteins within cells in response to CAP. Proteome and phosphorylome are included to investigate the functional activity of breast cancer cells in response to CAP treatment, which can be coupled together to identify critical proteins and phosphorylation sites altered after CAP treatment.

The lactylome and acetylome data include protein lactylation and acetylation quantification and modification sites that provide clues implicating metabolic reprogramming of breast cancer cells in response to CAP treatment. Given the competition between lactylation and acetylation on the posttranslational landscape of cells under dynamic physiological transitions or pathological conditions [40], integrative analysis of the lactylome and acetylome may identify vital molecules marking metabolic switch of transformed cells that are vulnerable to CAP treatment.

#### Sample type

Samples used for generating omics data currently contain cells and tumors from cell‐derived xenograft (CDX) mice model. The whole transcriptome, lactylome, and acetylome data were sequenced from cells, and the proteome and phosphorylome data were produced using animal materials. CDX models were included to ensure the accuracy of accessing the functionality and activity of the proteome by adopting an in vivo environment. Cells were used for generating other levels of omics data to ensure a stable reflection on signal fluctuations at multiple levels, especially when lactylome and acetylome are recommended to be interrogated together. The SUM159PT cells were used as the TNBC cell line, and MCF7 cells were adopted as the non‐TNBC cell control. For data sequenced using CDX models, no cell control was provided. Three replicates were included in each type of omics data.

#### Data organization

The data are organized in a hierarchical structure, with samples, genes/proteins, and modifications as the main nodes. Each node contains detailed information about the corresponding entity, such as expression levels, modification sites, and functional annotations. The database is constantly updated with new data to ensure the completeness and accuracy of the data resource.

### Web interface

Users can log in to the Hiplot official website (https://hiplot.com.cn), then click on “Tools” and “Data Analysis” to find and use the CAPmed‐BC service. For a better experience, users could click the full‐screen button. The web interface of CAPmed‐BC is designed to be intuitive and user‐friendly, allowing users to easily access and analyze the omics data. The interface includes the following main sections.

#### Home page

The home page provides an overview of the database, including the purpose of the database, the types of omics data available, and the main functions of the website. Users can quickly understand the scope and application of the database on the home page.

#### Data analysis page

The data analysis page provides a set of data analysis tools to help users perform in‐depth analysis of the omics data. The tools include differential expression analysis, GO and KEGG enrichment analyses, GSEA, and PPI network analysis. Users can select the desired data set and appropriate analytical tools for analysis. The results will be displayed in a visual and intuitive manner, allowing users to easily interpret the data and draw conclusions.

#### Help page and about page

The “Help” page is an educational hub for users of all levels, especially for beginners. With the help of this page, users could easily play with this database from having only basic concepts of the omics data and bioinformatics concepts. The “About” page provides detailed information about the website's support team and our previous work. Additionally, email addresses are provided to enable more direct communication, through which users can send their inquiries, such as possible errors with a particular tool, suggestions for new features, or general questions on the data or bioinformatics analysis.

### Data analysis tools and functionalities

The data analysis section of the website offers a range of tools for analyzing the omics data.

#### Differential expression analysis

Users can perform differential expression analysis for each type of omics data. For transcriptome data, users can compare gene expression levels between CAP‐treated and control samples of TNBC or non‐TNBC cells at different time points (e.g., 1 h vs. control, 8 h vs. control). For proteome and phosphorylome, users can perform the comparison between CAP‐treated and nontreated samples in TNBC cells, and characterize specific phosphorylated sites of particular interest. For lactylome and acetylome, users can identify critical sites subjected to differential lactylation and acetylation in response to CAP treatment, and specifically in TNBC cells as compared with non‐TNBC cells. The analysis results are presented as volcano plots, showing the fold change and statistical significance of each molecule.

#### GSEA

GSEA is used to determine whether a particular set of molecules is significantly enriched in a given biological function or pathway. The database provides several functional sets from databases such as GO. Users can perform GSEA using their differentially expressed molecules of interest. The results are presented as enrichment plots, indicating the significance of the enrichment and the leading molecules in the set for request.

#### GO and KEGG analysis

The website offers direct access to GO and KEGG pathway analysis. A list of differentially expressed molecules are used as the input, and the system will perform GO and KEGG enrichment analysis automatically. The results are shown in a hierarchical manner, with the enriched GO terms and KEGG pathways presented in a table and a bubble plot, along with their statistical significance and the number of molecules involved in each term/pathway. For both GSEA and GO/KEGG analysis, the R package clusterProfiler was used [43].

#### PPI network analysis

For proteome data, users can construct a PPI network analysis. The website integrates databases such as STRING [44] to retrieve protein–protein interaction information. Users can input a list of proteins and visualize their interaction network. The network can be customized, with options available to highlight differentially expressed proteins. The users can also change the colors of the nodes and edges, and adjust the layout.

### Examples of multi‐omics data analysis using CAPmed‐BC

**FIGURE 3:**
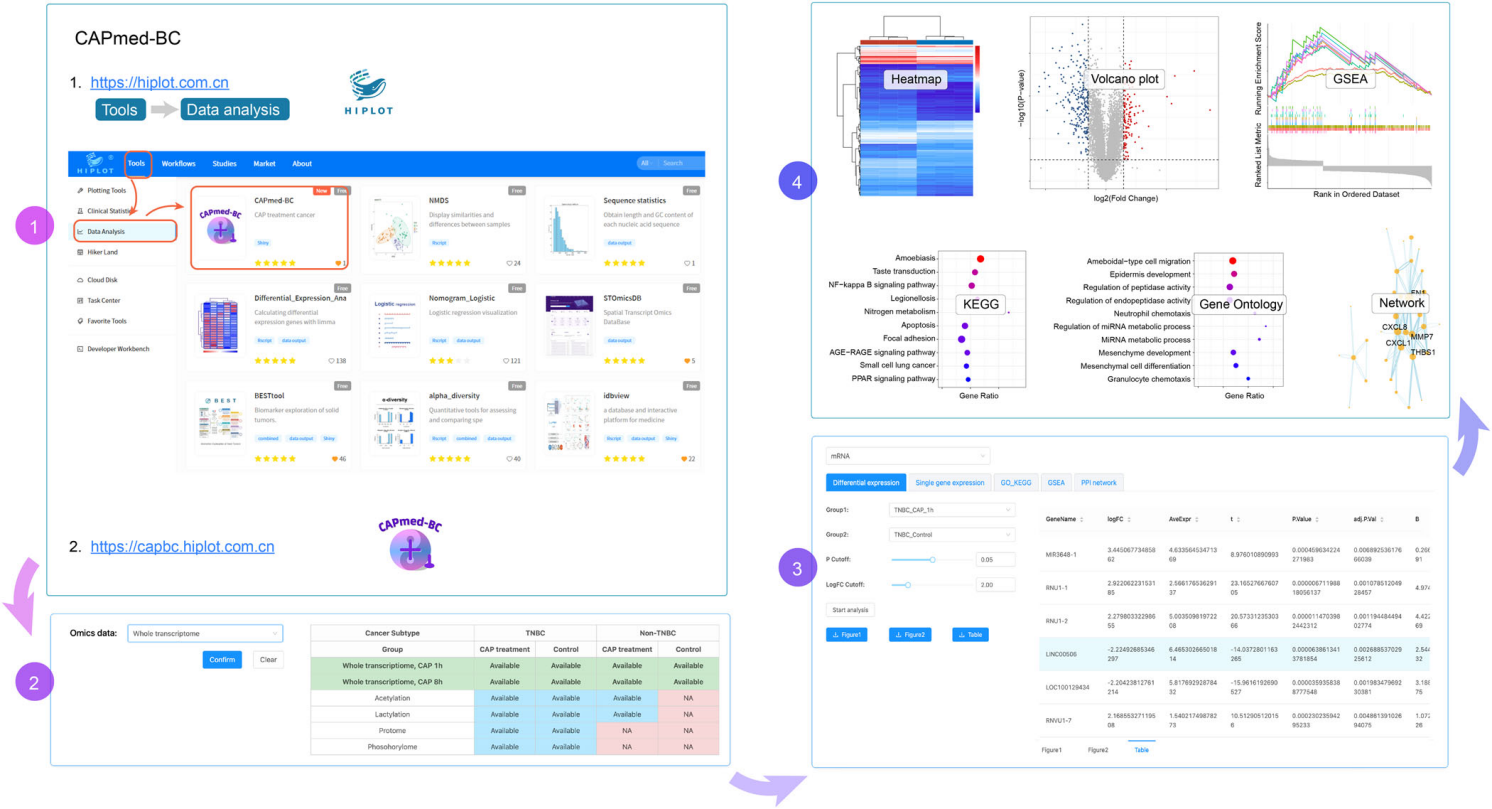
A demonstration of data analysis workflow in cold atmospheric plasma medicine in breast cancer (CAPmed‐BC). This figure illustrates how to use the CAPmed‐BC for interrogating the landscape of a particular omics in response to CAP from multiple perspectives, that is, differential expression, gene ontology (GO) and Kyoto Encyclopedia of Genes and Genomes (KEGG) enrichment, gene set enrichment analysis (GSEA) and protein–protein interaction (PPI) network. Steps are labeled with numbers on the right. Step 1: Access the URL. Step 2: Select the omics data. Step 3: Select the analysis tools and analysis parameters. Step 4: A demonstration of the outputs.

To demonstrate the functionality and utility of CAPmed‐BC, we present a case of multi‐omics data analysis using the database. In this case, we interrogated the instant mRNA transcriptome landscape of TNBC cells measured at 1 h post‐CAP exposure. We selected the “Whole transcriptome” data and clicked the “Confirm” button. The corresponding datasets used for this analysis were highlighted in green. Then, we selected the treated (“TNBC_CAP_1 h”) and control (‘TNBC_Control’) samples and chosen 0.05 and 2 as the “P Cutoff” and “LogFC Cutoff” thresholds, respectively, in the “Analysis” page. After clicking the “Start analysis” button, the heatmap showing differential expression, enriched GO terms and KEGG pathways, GSEA graph, and PPI network of the list of differentially expressed genes from the comparison groups will be automatically generated in each display window (Figure 3).

**FIGURE 4:**
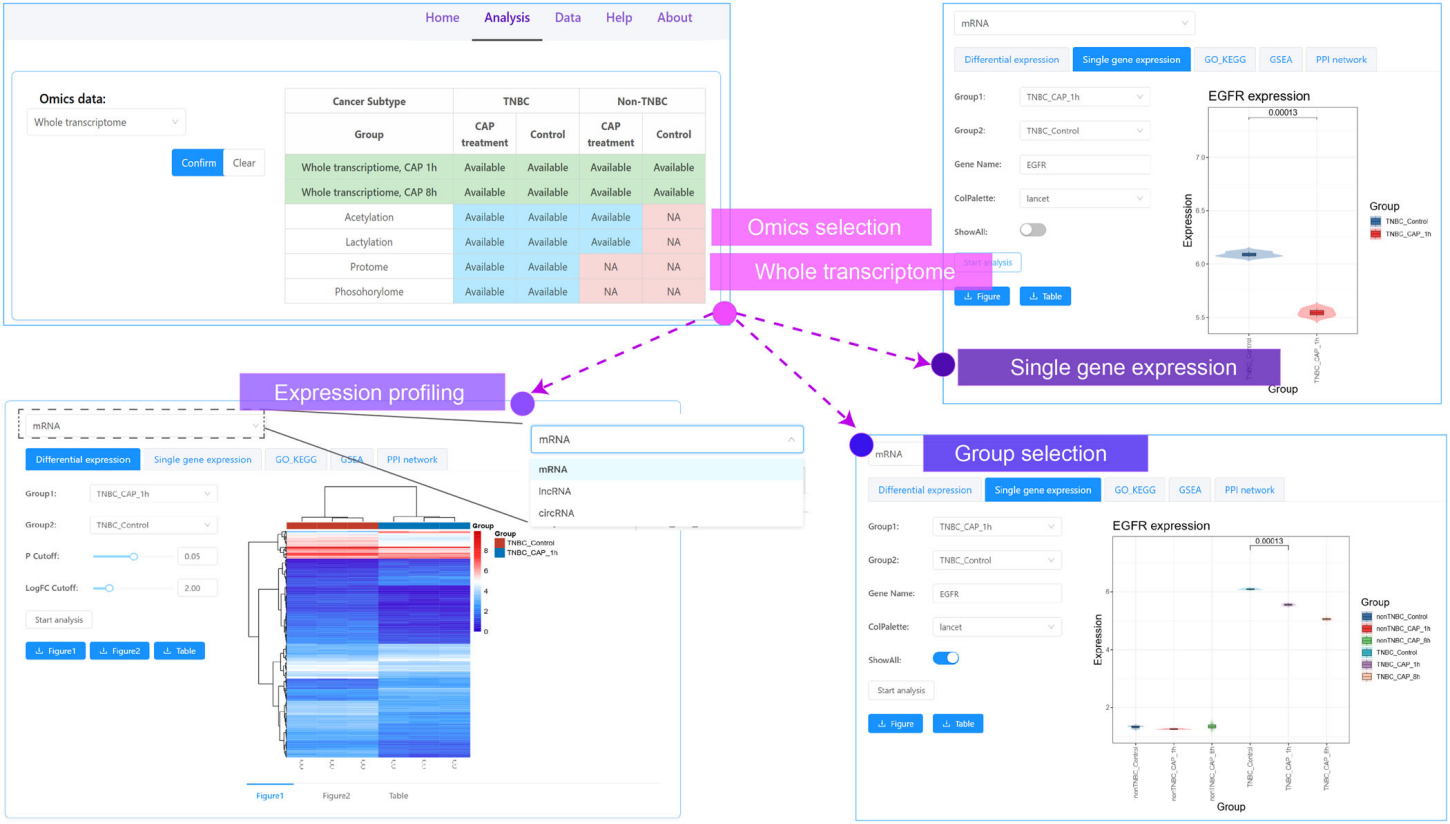
A demonstration of single‐gene expression analysis using whole transcriptome data in cold atmospheric plasma medicine in breast cancer (CAPmed‐BC). This figure illustrates how to use CAPmed‐BC to analyze a particular gene of interest. As the whole transcriptome is the sole omics data that contains information on time series, the “ShowAll” button offers the option for viewing the dynamic profile of a particular molecule in all samples.

For a particular gene of interest, one can perform single‐gene expression analysis. Here, we were interested in interpreting the expression levels of *EGFR*. By clicking the “Single gene expression” button and inputting the gene symbol in “Gene Name”, we obtained violin plot for the expression of this gene in “TNBC_CAP_1h” and “TNBC_Control” groups with the *p*‐value being marked. By sliding the “ShowAll” button to the right‐hand side and rerun the analysis, the expression levels of this gene in all samples (i.e., nonTNBC cell control, nonTNBC cell 1 h‐CAP postexposure, nonTNBC cell 8 h‐CAP postexposure, TNBC cell control, TNBC cell 1 h‐CAP postexposure, TNBC cell 8 h‐CAP postexposure) were displayed. The results showed that EGFR mRNA level was higher in TNBC cells than in nonTNBC cells, and CAP significantly reduced the transcriptional level of *EGFR,* and the reduction level enhanced with the posttreatment duration under the observation time scale (Figure 4).

**FIGURE 5:**
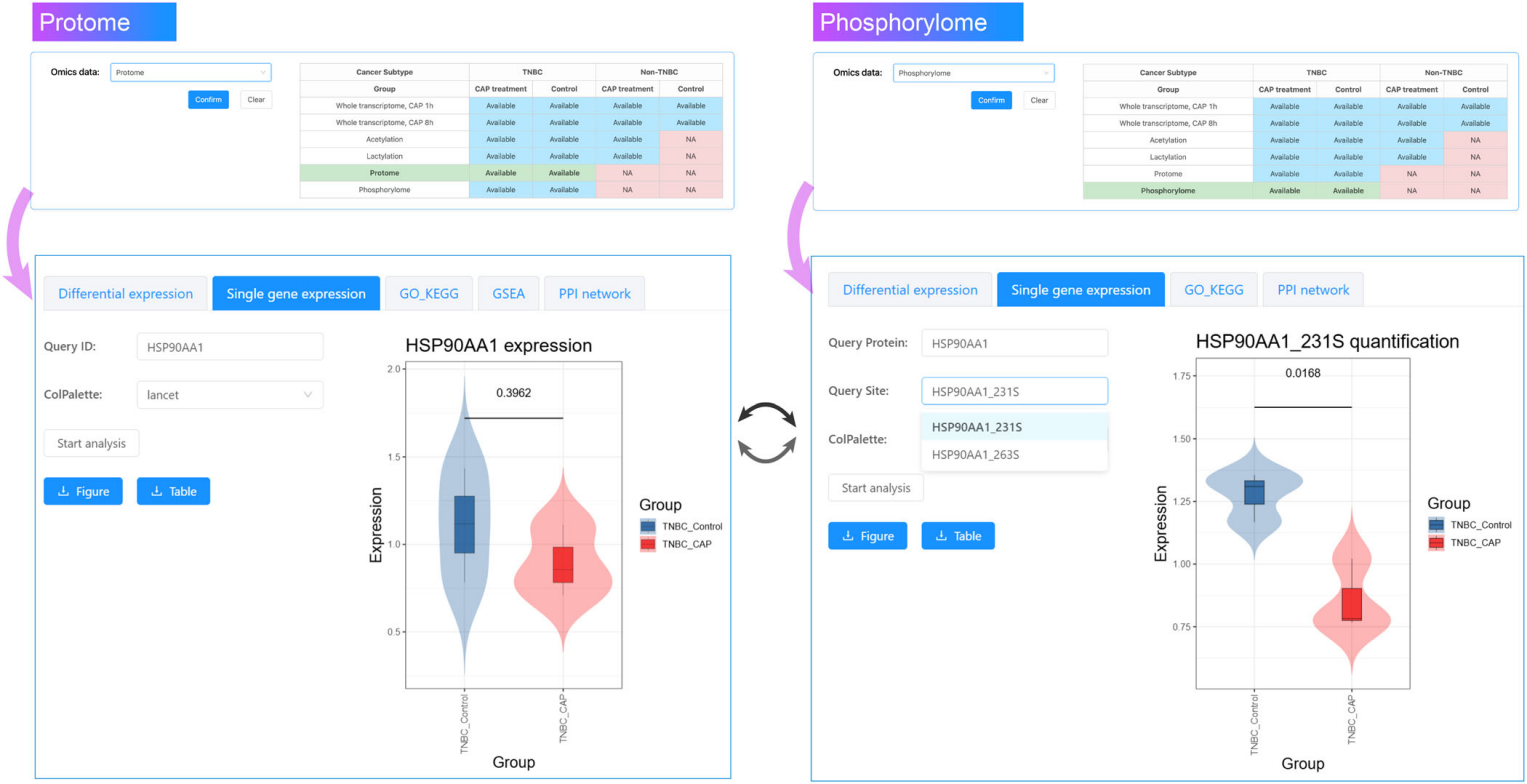
A demonstration of combinatorial analysis using proteome and phosphorylome data in cold atmospheric plasma medicine in breast cancer (CAPmed‐BC). This figure illustrates how to differentiate the use of CAP on protein amount and functionality using CAPmed‐BC. As the proteome and phosphorylome share the same in vivo sample source and reflect distinct perspectives of cellular functionalities, their combined use may help improve the analysis resolution to the level of, e.g., protein stability, activity, and cellular localization.

Next, we examined the role of CAP on the amount and activity of HSP90AA1. To do so, we selected “Protome” in the “Analysis” page, and inputted “HSP90AA1” as the “Query ID” in the “Single gene expression” panel. After clicking “Start analysis”, we obtained the violin chart of the protein levels of HSP90AA1 in TNBC cells treated with CAP or not. By performing a similar operation using “Phosphorylome” as the omics data, and selecting HSP90AA1_231S as the “Query Site”, we obtained quantified phosphorylation levels of this site in CAP‐treated and control TNBC samples. The results showed significant reduction on HSP90AA1(231S) phosphorylation but not the level of this protein (Figure 5), suggesting that CAP functions by arresting the functionality but not its total amount.

**FIGURE 6:**
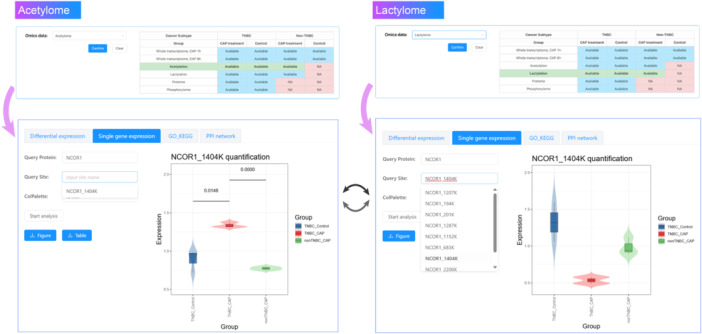
A demonstration of combinatorial analysis using acetylome and lactylome data in cold atmospheric plasma medicine in breast cancer (CAPmed‐BC). This figure illustrates how to identify metabolic clues for interrogating the molecular mechanisms of TNBC cells in response to CAP using CAPmed‐BC. Since opposite lactylation and acetylation profiles may mark the fate of pyruvate and implicate metabolic alteration of cells in response to CAP, the acetylome and lactylome stored in CAPmed‐BC can be used in couple to identify vital molecules characterizing metabolic reprogramming caused by CAP.

As the last and most important example, we selected “Acetylation” as the omics data, and focused on the protein nuclear receptor corepressor 1 (NCOR1). By inputting “NCOR1” in the “Query Protein” box in the “Single gene expression” panel, we found that NCOR1_1404 K was the sole acetylation site specifically identified from TNBC cells (i.e., not identified from non‐TNBC cells) after CAP treatment. By confirming this site and clicking the “Start analysis” button, we obtained the acetylation profiles of NCOR1(1404K) site in TNBC control cells, CAP‐treated TNBC cells, and CAP‐treated non‐TNBC cells. We performed a similar operation using the “Lactylation” omics. Among all available lactylation sites in the omics data, we selected “NCOR1_1404K”, and obtained the lactylation profiles of NCOR1 (1404K) site in all samples. The results showed opposite acetylation and lactylation profiles of NCOR1 (1404K) in CAP‐treated TNBC cells as compared with the untreated control and nonTNBC control (Figure 6). This protein and post‐modification site could be considered as a molecular clue for interrogating the metabolism of TNBC cells in reaction to CAP treatment.

## CONCLUSION

As a focus‐driven tumor‐specific module of Hiplot, CAPmed‐BC is the first omics data resource and an analytical platform detailing the response of cancer cells to CAP treatment. It provides data storage, processing, and analysis for breast cancer research and plasma oncology. It not only provides basic analysis such as differential expression analysis, GO and KEGG enrichment, GSEA, and PPI network, but also serves as an integration tool to unveil the dynamic behavior as well as altered activity and metabolism of cancer cells in response to CAP perturbation. The practical and user‐friendly interface of CAPmed‐BC provides users with an easy‐to‐use and hands‐on tool to quickly interrogate these CAP‐based omics data for clues implicating the efficacy and specificity of CAP in treating TNBC cells, possible biomarkers indicative of cells' sensitivity to CAP treatment, and even the mechanism driving TNBC pathogenesis. CAPmed‐BC is the only database designed for CAP‐associated studies. Its emergence, the multi‐omics data stored, the carefully designed data integration strategy and rational, as well as the easy‐to‐navigate interface, will substantially advance the field of plasma medicine and, in particular, plasma oncology. Also, it will accelerate the in‐depth understanding of TNBC, and the anticancer mechanisms of CAP that can be easily extended to other types of malignancies.

Data included in CAPmed‐BC demonstrated the differences across various omics in TNBC cells before and after CAP treatment. Besides analyzing data at each omics level, a joint analysis of multi‐omics data could potentially provide more in‐depth integration and greater insights into the underlying mechanisms. We suggested three ways of data analysis or combination with examples in this study, that is, analyzing the whole transcriptome to learn the short‐ and long‐term dynamic fluctuation of TNBC cells in response to CAP, combining the proteome and phosphorylome to investigate functional variations of TNBC cells after CAP treatment, and coupling the lactylome and acetylome to explore metabolic clues leading to the therapeutic efficacy of CAP in treating TNBC cells. These suggested combinations reflect the rational on the selection of data sources included in CAPmed‐BC that could generate unique results for interpretation. Yet, it does not limit the users from choosing other levels of omics data to obtain their desired outcomes.

With our incremental understandings on the types of omic profiles existing within cells and their functionalities, especially at the posttranslational modification levels, more high‐throughput technologies will be established. Thus, we will continue to update CAPmed‐BC and add more levels of omics data, such as chromatin immunoprecipitation sequencing (ChIP‐seq) and assay for transposase‐accessible chromatin with high‐throughput sequencing (ATAC‐seq) to substantiate its omics diversity and deepen the investigation in‐depth. Also, advances in single‐cell sequencing have made it possible to investigate the behavior of cells at multiple omic levels at the resolution of single cells. Therefore, we will consider supplementing CAPmed‐BC with single‐cell omics data to enhance its analytical resolution. In the meanwhile, we will develop *de novo* algorithms and establish self‐adapted tools to integrate these datasets that we believe may advance many other research fields beyond plasma medicine and oncology.

The development of CAPmed‐BC marks a pivotal advancement of Hiplot, making it not only an analytical platform but also a data storage source with specific focuses. We anticipate that functional modules such as CAPmed‐BC can help expedite the clinical translation of innovative tools such as CAP for the benefits of a greater number of cancer patients, which is far beyond the scope of identifying makers or drug targets.

## METHODS

### Sample preparation

Breast cancer cell lines, including SUM159PT and MCF7, were used for preparing in vitro cell or CDX in vivo samples. Both cells were purchased from Nanjing Kemo Biomedical Co., Ltd., which were available from American Type Culture Collection. Cells were authenticated using Short Tandem Repeat analysis following the instructions described in 2012 in ANSI Standard (ASN‐0002). SUM159PT cells were cultured using F12 (#P2090359, Damas) supplemented with 5% FBS (#FSP500, ExCellBio), 0.325% insulin, 1% N‐2‐hydroxyethylpiperazine‐N‐2‐ethane sulfonic acid, 0.0276% hydrocortisone, and 1% penicillin‐streptomycin (#BL505A, Biosharp). MCF7 cells were cultured using DMEM (#RNBK4719, SIGMA) supplemented with 10% FBS and 1% penicillin‐streptomycin. Both cells were cultured under 37°C, 5% CO_2_.

A self‐made CAP source, namely HighPen, was used to prepare CAP‐treated samples. The CAP source is composed of a controlled power supply, helium (He) gas cylinder, rotameter, and plasma jet that can work in a controllable high‐throughput fashion (Figure 2). The peak‐to‐peak voltage applied to the electrode was set in the range of 0.96–1.24 KV, the sine wave frequency was set to 10 kHz, the gas flow rate was set to 1 L/min, and the distance between the CAP source and the dielectric surface was fixed to 13 mm. CAP‐activated medium was used to treat cells, which was prepared by setting the distance between the CAP nozzle and the media surface to 13 mm, the peak‐to‐peak electrode voltage to 1.1 KV, the sine wave frequency to 8.8 KHz, and the gas flow rate to 1 L/min.

For in vitro sample preparation, 2 mL of the cell culture medium in a 12‐well plate was exposed to CAP for 4 min, which was used to replace the cell culturing medium in the CAP‐treated group. The 1 h and 8 h post‐CAP treatment groups in the whole transcriptome data were cells incubated with CAP‐activated medium for 1 h and 8 h, respectively.

For in vivo sample preparation, cancer cells suspended in PBS were subcutaneously injected into the right forelimbs of 4‐week‐old female nude BALB/c mice. The initial weight per mouse was 21 ± 2 g. Each mouse was injected with 1 × 10^6^ cells. After 2 weeks feeding, mice with tumors grown to 5 ± 0.5 mm in diameter were recruited. BALB/c mice inoculated with cancer cells were randomly grouped. Mice were treated every 2 days till the 15th day after the recruitment. During each operation, mice were pre‐anesthetized intraperitoneally with ketamine (10 mg/mL), with the injection volume being 10 μL/g of the mouse weight.

CAP‐activated PBS or PBS was subcutaneously injected into two spots of each tumor‐carrying mouse with 100 μL/spot for the treatment and control groups. Mice were killed on the 18th day starting from the initial date of recruitment, and the tumors were dissected. All animal experiments were performed in accordance with the Guidelines for Nursing and Utilization of Experimental Animals issued by the National Institutes of Health and approved by the Animal Experiment Center of Jiangnan University under IACUC Issue JN. NO2021121560900325[573]

As the final step of sample preparation, cells were harvested promptly following previously established protocols. For omics analysis, appropriate lysis buffers were used to extract total RNA or proteins for transcriptome, proteome, phosphorylome, acetylome, and lactylome analysis. Protease and phosphatase inhibitors were added to the lysis buffers as necessary to prevent protein degradation and maintain the integrity of posttranslational modifications.

### Omics data collection

The omic profiles were sequenced at Jingjie PTM BioLab (Hangzhou) Co. Inc.

#### Whole transcriptome

Total RNA was isolated from the treated and control cells using TRIzol reagent according to the manufacturer's instructions. The quality and quantity of the RNA were measured using a NanoDrop spectrophotometer and agarose gel electrophoresis. For mRNA, lncRNA, and circRNA sequencing, library preparation was performed using the NEBNext Ultra II RNA Library Prep Kit. The RNA samples were fragmented, and cDNA was synthesized using random primers. For mRNA, polyA selection was employed to enrich the mRNA fraction. For lncRNA and circRNA, rRNA depletion was carried out to increase the proportion of noncoding RNAs in the sequencing library. After size selection, the libraries were amplified by PCR. The prepared libraries were sequenced using an Illumina NovaSeq platform to generate raw sequencing reads.

#### Proteome

Proteins were extracted from the cell lysates using the RIPA buffer‐based extraction protocol. After protein quantification, samples were digested using trypsin to generate peptide fragments. These peptides were then labeled with Tandem Mass Tag labeling reagents for quantitative proteomics analysis. Labeled peptides were separated by liquid chromatography and analyzed by liquid chromatography‐tandem mass spectrometry (LC‐MS/MS).

#### Phosphorylation

Phosphopeptides were enriched from the digested peptide mixtures using immobilized metal ion affinity chromatography. The enriched phosphopeptides were then analyzed by LC‐MS/MS with optimized parameters for detecting phosphorylated peptides.

#### Lactylation

Lactylated peptides were enriched using specific antibodies against lactylated lysine residues. The enrichment process involved incubating the peptide mixtures with the antibody‐conjugated beads, followed by washing and elution of the bound lactylated peptides. Eluted peptides were analyzed using LC‐MS/MS following a similar protocol with phosphorylation analysis.

#### Acetylation

Acetylated peptides were enriched using acetyl‐specific antibody‐based methods. Peptide samples were incubated with antibody‐coated beads, and acetylated peptides were eluted and analyzed by LC‐MS/MS after thorough washing.

### Data preprocessing

#### Whole transcriptome

For mRNA, lncRNA, and circRNA sequencing data, the raw reads were first subjected to quality control using FastQC. Low‐quality reads, adapter‐contaminated reads, and reads with a high proportion of N bases were removed using the software Trimmomatic. The remaining clean reads were then aligned to the reference genome (e.g., human genome hg38) using the alignment tool TopHat2 for mRNA and the tool STAR for lncRNA and circRNA. After alignment, gene expression levels were quantified using Cufflinks for mRNA and specialized tools for lncRNA and circRNA quantification [45, 46].

#### Proteome

The raw MS data from proteome analysis were processed using the software MaxQuant. The peptide spectra were searched against UniProt [47] human to identify peptides and proteins. Parameters used for searching included enzyme specificity (e.g., trypsin with specified cleavage rules), mass tolerance for precursor and fragment ions, and consideration of variable and fixed modifications. Protein quantification was performed based on the intensity of peptide signals, and data normalization was performed to account for differences in sample loading and instrument response.

#### Phosphorylation, lactylation and acetylation

For phosphorylation data, the MS/MS spectra were analyzed using MaxQuant. The identification of phosphopeptides was based on the presence of phosphorylated amino acid residues (i.e., serine, threonine, or tyrosine) and the corresponding mass shifts. The quantification of phosphorylation levels was calculated relative to the total amount of the corresponding peptide or protein. Data filtering was applied to remove phosphorylated peptides of low confidence.

The analysis of lactylome and acetylome data followed a similar workflow to phosphorylome analysis. The MS/MS datum were processed to identify lactylated or acetylated peptides followed by quantification. After removing peptides of low confidence, identified lactylated or acetylated peptides were mapped to their corresponding proteins.

### Website implementation

Based on Hiplot web application protocols, the website was developed using a combination of web technologies. The backend infrastructure was built using Python‐based frameworks, that is, Django, which provided a robust and scalable platform for handling data storage, processing, and user requests. The database management system, PostgreSQL, was used to store the omics data, including gene expression values, protein abundances, and modification levels. For the front‐end interface, HTML, CSS, and JavaScript were used to create an intuitive and user‐friendly interface. Interactive visualizations were implemented using libraries like Plotly, which enabled users to explore the omics data in a dynamic way. The website was designed to be responsive, ensuring that it could be accessed and used on various devices.

## AUTHOR CONTRIBUTIONS

**Xiaofeng Dai**: Conceptualization; investigation; funding acquisition; writing—original draft; writing—review and editing; visualization; validation; methodology; software; formal analysis; project administration; resources; supervision; data curation. **Mingjie Wang**: Project administration. **Yang Liu**: Validation.

## CONFLICT OF INTEREST STATEMENT

The authors declare no conflict of interest.

## ETHICS STATEMENT

All animal experiments were performed in accordance with the Guidelines for Nursing and Utilization of Experimental Animals issued by the National Institutes of Health and approved by the Animal Experiment Center of Jiangnan University (No. JN. NO2021121560900325[573]).

## Data Availability

The data that supports the findings of this study are available in the supplementary material of this article. The raw data have been deposited in the National Genomics Data Center, China National Center for Bioinformation/Beijing Institute of Genomics, Chinese Academy of Sciences, with the accession number being OMIX009850 and the web link being https://ngdc.cncb.ac.cn/omix/release/OMIX009850. The data and scripts used are saved in GitHub at https://github.com/mingjiewang/CAPmed-BC. Supplementary information (graphical abstract, slides, videos, Chinese translated version, and update materials) may be found in the online DOI or iMeta Science http://www.imeta.science/.
